# Cardiovascular Effects of Tourniquet Application with Cardiac Cycle Efficiency: A Prospective Observational Study

**DOI:** 10.3390/jcm13102745

**Published:** 2024-05-07

**Authors:** Merve Seker, Serap Aktas Yildirim, Halim Ulugol, Bulent Gucyetmez, Fevzi Toraman

**Affiliations:** Department of Anesthesiology and Reanimation, Acibadem Mehmet Ali Aydinlar University School of Medicine, Istanbul 34752, Turkey; serapaktas79@yahoo.com.tr (S.A.Y.); halimulugol@yahoo.com.tr (H.U.); bulentgucyetmez@gmail.com (B.G.); ftoraman@gmail.com (F.T.)

**Keywords:** cardiac cycle efficiency, arterial elastance, cardiac contractility, tourniquet

## Abstract

**Objectives:** The impact of the tourniquet on cardiac efficiency remains unknown. This study aimed to assess the impact of the tourniquet on cardiac cycle efficiency (CCE) and to interpret how general anesthesia (GA) or combined spinal epidural anesthesia (CSEA) affects this during surgery using cardiac energy parameters. **Methods:** This prospective observational study included 43 patients undergoing elective unilateral total knee arthroplasty (TKA) with a tourniquet divided into GA (*n* = 22) and CSEA (*n* = 21) groups. Cardiac energy parameters were measured before anesthesia (T1), pre-tourniquet inflation (T2), during inflation (T3–T8), and post-deflation (T9). The estimated power of the study was 0.99 based on the differences and standard deviations in CCE at T2–T3 for all patients (effect size: 0.88, alpha error: 0.05). **Results:** CCE decreased significantly more at T3 in the GA group than in the CSEA group, whereas dP/dt*_max_* and Ea increased more (*p* < 0.05, *p* < 0.001, and *p* < 0.01, respectively). At T9, CCE increased significantly in the GA group, whereas dP/dt*_max_* and Ea decreased (*p* < 0.05, *p* < 0.001, and *p* < 0.001, respectively). **Conclusions:** The tourniquet reduces cardiac efficiency through compensatory responses, and CSEA may mitigate this effect.

## 1. Introduction

In total knee arthroplasty (TKA), a tourniquet is used to optimize the surgical field and reduce bleeding [1]. Inflation of the tourniquet may lead to increased blood pressure due to preload and afterload elevation, and vice versa for deflation [2,3,4,5]. Even fatal cardiac events have been reported after tourniquet deflation [6,7]. Therefore, close hemodynamic monitoring is essential to manage potential cardiac adverse events related to tourniquet application. Various methods, such as transesophageal echocardiography, esophageal doppler, and pulmonary artery catheterization are used for detailed monitoring of cardiovascular changes during anesthesia procedures [8,9,10]. However, less invasive methods based on pulse wave analysis have started to be preferred [11].

Cardiac parameters such as arterial elastance (Ea), dP/dt*_max_*, cardiac cycle efficiency (CCE), and cardiac power output (CPO) can be detected by using minimally invasive cardiac monitoring that uses pulse wave analysis [12,13,14,15,16]. CCE, among these, provides a global assessment of total cardiovascular performance by demonstrating that the heart is working outside its efficiency limits due to the use of compensatory mechanisms that cause an increase in cardiac workload [12]. But still, we have limited data on how cardiac function is altered in tourniquet-applied TKA.

Our recent study demonstrated that CCE was affected by Trendelenburg position in general anesthesia (GA) [16]. Thus, it is expected that using a tourniquet can dramatically change CCE. Investigation of arterial waveform characteristics specific to tourniquet-applied total knee arthroplasty may help to develop preventive strategies against possible complications. For this reason, this study’s primary aim is to investigate the changes in CCE after performing a tourniquet in TKA. The secondary aim is to investigate how general anesthesia (GA) or combined spinal epidural anesthesia (CSEA) affects this.

## 2. Materials and Methods

### 2.1. Ethics Approval

This prospective, observational study, approved by the Acibadem Mehmet Ali Aydinlar University Local Ethics Committee, Istanbul, Turkey on 2 September 2022 (Decision No: 2022-14/12), was conducted between September 2022 and April 2023 in Altunizade Acibadem Hospital.

### 2.2. Trial Registration

The trial was registered (No: NCT06158165) on 5 December 2023.

### 2.3. Patients

The study included 43 individuals aged 18 years and above with American Society of Anesthesiology (ASA) physical status 1–3, who underwent elective unilateral total knee arthroplasty (TKA) using a tourniquet at Altunizade Acibadem Hospital. The monitoring method and timing were determined by the attending anesthesiologist for each patient, and patients were included who were planned to have an arterial cannula inserted before anesthesia induction according to comorbidity and major surgery. Patients younger than 18 years, those with arrhythmia, heart failure, valve disorder, myocardial infarction history in recent times, and non-consenting individuals were excluded from the study. Informed consent was obtained from all patients. This research is in accordance with the STROBE (STrengthening the Reporting of OBservational studies in Epidemiology) statement. Demographic data and comorbidities were recorded in the anesthesia outpatient clinic. After the participants were informed about the general anesthesia (GA) and combined spinal epidural anesthesia (CSEA) techniques in the pre-operative interview, the anesthesia method was determined according to the patients’ preferences. Based on this distinction, patients were categorized into GA (*n* = 22) and CSEA (*n* = 21) groups.

### 2.4. The PRAM Parameters

CCE, which is used to evaluate the effect of the tourniquet on the cardiovascular system, is calculated by the formula W(t)sys/W(t)beat × K(t), reflecting the ratio of the energy spent by the heart in systole during each beat to the energy spent during the total cycle (systole + diastole), and this provides indirect information about the cardiac efficiency and the use of compensation mechanisms [13]. This parameter can range from +1 (ideal condition with no energy expenditure) to negative values. The greater the energy expended to generate a given SV, the lower the CCE value. A decrease in CCE to negative values indicates that compensation mechanisms are activated and cardiac efficiency decreases [12]. CCE, which is also associated with ventriculo-arterial coupling (VAC), makes it possible to evaluate the vascular and cardiac systems together on the basis of energy use [14]. CPO is a parameter obtained by multiplying mean arterial pressure (MAP) by cardiac output (CO) (CPO (W) = MAP × CO/451) and is considered an indicator of left ventricular stroke work (LVSW) [17]. The physiological range at rest is 0.80–1.20 Watts [17]. CPO, which includes both pressure and flow parameters (binary factors), provides information to clinicians about cardiac reserve and prognosis [18,19]. Ea is obtained from the ratio of left ventricular end-systolic pressure (LVESP) to SV and represents diverse arterial characteristics, encompassing outflow resistance, compliance, and wall stiffness [20,21,22,23]. The physiological range for Ea is 1.10–1.40 mmHg mL^−1^ at rest [23]. dP/dt*_max_* depends on the relationship between left ventricular function and arterial tone and is generally used as an expression of myocardial contractility [15,24]. The physiological range for dP/dt*_max_* is 0.9–1.3 mmHg s^−1^ at rest [23].

### 2.5. Study Protocol

#### 2.5.1. Anesthesia

After ensuring sterile conditions, radial artery cannulation was conducted with a 20-gauge catheter under local anesthesia.

In the group receiving general anesthesia, anesthesia induction was performed with intravenous (IV) 2 mg/kg propofol, IV 1 μg/kg remifentanil, and IV 0.6 mg/kg rocuronium. After intubation, the tidal volume was set to 6–8 mL/kg and the respiratory rate was modified to reach an end tidal carbon dioxide of 30–40 mmHg. General anesthesia was sustained through inhalation of sevoflurane (1.5–2%) and IV infusion of remifentanil (0.02–0.5 μg/kg/min). Hypnotics and opioids were adjusted according to the patient’s hemodynamic response, and targeted bispectral index (BIS) values (Covidien, Boulder, CO, USA) were in the range of 40–50 for depth of anesthesia.

In the group undergoing CSEA, an epidural puncture was executed using an 18-gauge Tuohy needle with the ‘loss of resistance’ technique, approaching the midline from the lumbar region of L4–L5. An 18-gauge Tuohy needle was used to place a 27-gauge needle through which 15 mg 0.5% hyperbaric bupivacaine and 20 µg fentanyl were administered into the subarachnoid space. Finally, a 20-gauge catheter was placed in the epidural space in a cephalic direction. The participants were positioned in supine posture and checked by performing a pin-prick test every 2 min until the targeted block level reached T10. Subsequently, an IV infusion of propofol at a rate of 50–100 µg/kg/min was initiated and titrated to achieve a BIS level below 75. No additional epidural dose was required for any patient until the completion of surgery.

The tourniquet was inflated 10 min after induction of anesthesia for all patients. The pressure level of the tourniquet was set to 250 mmHg, with a maximum duration of 2 h.

#### 2.5.2. Fluid Administration

All patients brought to the operating room were started on intravenous (IV) Ringer’s lactate solution at a dose of 5 mL/kg/h before anesthesia induction as a standard. If the SAP value was below 30% of the pre-anesthetic level, it was deemed hypotension, and ephedrine was administered. All the surgical procedures were performed by the same team.

#### 2.5.3. Hemodynamic Monitoring

Stroke volume index (SVI), systolic arterial pressure (SAP), CCE, Ea, dP/dt*_max_*, and CPO values were monitored using the MostCare (Vytech, Vygon, Padua, Italy) device operating with the PRAM method. Hemodynamic and PRAM parameters were documented 1 min before anesthesia induction (T1), 10 min after anesthesia induction, which is 1 min before tourniquet inflation (T2), 1 min after tourniquet inflation (T3), 10 min after inflation of the tourniquet (T4), 20 min after inflation of the tourniquet (T5), 30 min after inflation of the tourniquet (T6), 60 min after inflation of the tourniquet (T7), 1 min before deflation of the tourniquet (T8), and 1 min after deflation of the tourniquet (T9).

### 2.6. Statistical Analysis

Data were given as mean (standard deviation), median (quartiles), and percentages. Kolmogorov–Smirnov and Shapiro–Wilk tests were used to detect normal distribution. Wilcoxon rank and Paired Student *t*-tests were used for comparisons of PRAM parameters between time points, whereas Student *t*, Mann–Whitney U, and chi-square tests were used for comparisons between GA and CSEA groups. For this study, two estimated powers were calculated. The first estimated power was 0.99, based on the differences in means and standard deviations of the CCE measurements at T2 and T3 for all patients (*t*-test for two dependent matched pairs; effect size: 0.88, sample size: 43, alpha error: 0.05, GPower 3.1.9.4 version). The second estimated power was 0.96, based on the differences in means and standard deviations of the delta-CCE of both groups (*t*-test for two independent groups; effect size: 1.0, sample sizes: GA = 22 and CSEA = 21, alpha error: 0.05, GPower 3.1.9.4 version). SPSS version 29.0 was used for all statistical analyses, and a *p*-value below 0.05 was regarded as statistically significant.

## 3. Results

**Figure 1:**
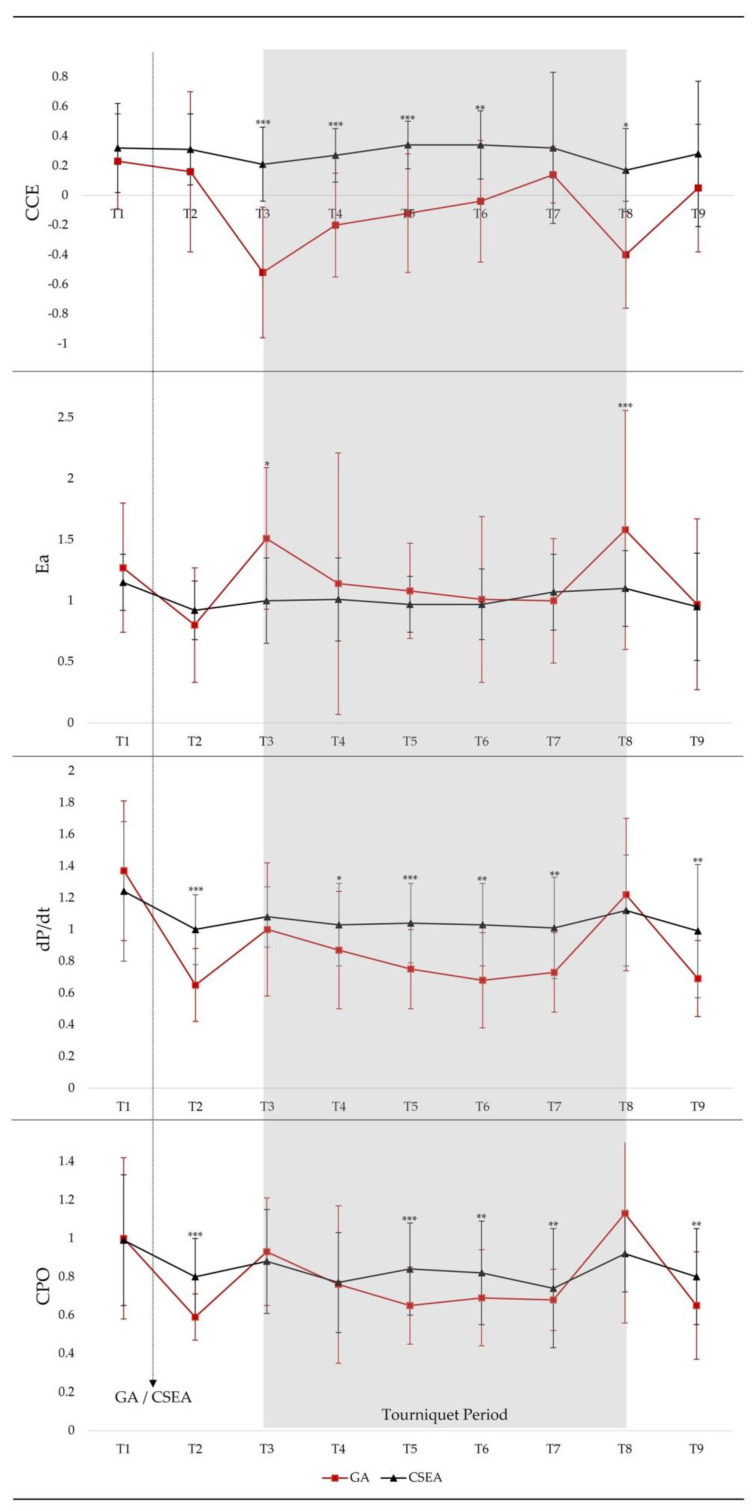
Comparisons between CCE, Ea, dP/dt, and CPO levels of groups in different times. GA, general anesthesia; CSEA, combined spinal epidural anesthesia. *, *p* = 0.05; **, *p* = 0.01–0.001; ***, *p* < 0.001.

Forty-three patients (GA: 22 and CSEA: 21) were included in this study. The mean value of the age for all patients was 70 years. The ASA score was 2 or higher in 88% of the patients. The two most common comorbidities were hypertension (72%) and diabetes mellitus (44%) (Table 1). Demographic data, comorbidities, preoperative ejection fractions (EF), pre-anesthetic induction PRAM parameters, and tourniquet duration were similar between the GA and CSEA groups (Table 1 and Figure 1).

For all patients at T3, SAP, Ea, dP/dt*_max_*, and CPO were significantly higher, whereas CCE was significantly lower than at T2 (*p* < 0.001 for all) (Table 2). On the contrary, at T9, SAP, Ea, dP/dt*_max_*, and CPO were significantly lower, whereas CCE was significantly higher than T8 (*p* < 0.001, *p* < 0.001, *p* < 0.001, *p* < 0.001, and *p* = 0.024, respectively) (Table 2). SVI was similar both after tourniquet inflation and deflation (*p* = 0.167 and *p* = 0.096) (Table 2).

Similarly, in the GA group at T3, SAP, Ea, dP/dt*_max_*, and CPO values were significantly higher, whereas CCE was significantly lower than at T2 (*p* < 0.001 for all) (Table 2). As for at T9, SAP, Ea, dP/dt*_max_*, and CPO values were significantly lower, whereas CCE was significantly higher than at T8 (*p* < 0.001, *p* < 0.001, *p* < 0.001, *p* < 0.001, and *p* = 0.024, respectively) (Table 2). SVI was similar both after tourniquet inflation and deflation (*p* = 0.259 and *p* = 0.106) (Table 2).

In the CSEA group at T3, SAP, Ea, dP/dt*_max_*, and CPO values were significantly higher, whereas CCE was significantly lower than at T2 (*p* < 0.001, *p* < 0.001, *p* = 0.003, *p* = 0.043, and *p* < 0.001, respectively) (Table 2). At T9, only SAP and CPO were significantly lower than at T8, whereas Ea, dP/dt*_max_*, and CCE were similar (*p* < 0.001, *p* = 0.008, *p* = 0.118, *p* = 0.369, and *p* = 0.375, respectively) (Table 2). SVI was similar both after tourniquet inflation and deflation (*p* = 0.445 and *p* = 0.055) (Table 2).

At T3, in the GA group, increases in SAP, Ea, dP/dt*_max_*, and CPO were significantly higher, whereas a decrease in CCE was significantly higher than in the CSEA group (*p* < 0.001, *p* = 0.042, *p* < 0.001, *p* < 0.001, and *p* < 0.001, respectively) (Table 3). At T9, decreases in SAP, Ea, dP/dt*_max_*, and CPO were significantly higher than in the CSEA group, whereas changes in CCE were similar in both groups (*p* < 0.001, *p* = 0.002, *p* < 0.001, *p* < 0.001, and *p* = 0.061) (Table 3). Changes in SVI at T3 and T9 were similar in both groups (*p* = 0.626 and *p* = 0.588) (Table 3). Furthermore, in the GA group, CCE values at T2, T3, T4, T5, T6, and T8 were significantly lower than in the CSEA group (Figure 1).

## 4. Discussion

Based on available information, this is the first research to evaluate the effect of a tourniquet on cardiovascular functions with PRAM parameters in elective unilateral TKA surgeries. In this study, it was demonstrated that pressure–volume changes caused by the tourniquet reduce cardiac efficiency through increased vascular tone (Ea) and changes in contractility (dP/dt*_max_*), independent of the anesthesia method, but this effect was attenuated by the CSEA method.

Tourniquet inflation induces sympathetic activation through pain and mediators released from vascular endothelium [25,26,27,28]. The release of norepinephrine as a result of sympathetic activation is known to increase cardiac contractility through beta-adrenergic receptors and to increase peripheral vascular resistance, hence increasing SAP, through alpha-adrenergic receptors [29,30,31]. This increase in SAP leads to an elevation in arterial system resistance and tension (arterial elastance), consequently increasing the total arterial load on the left ventricle (LV) [20]. In this study, SAP increased in both the GA and CSEA groups. This rise was more pronounced in the GA group. In both groups of patients, the increase in Ea value (LVESP/SV) without a change in SVI indicated an elevation in LVESP, demonstrating an increase in aortic impedance. Although attempts were made to balance the increase in LVESP with an increase in contractile strength (reserve), the sustainability of this situation depends on the contractile reserve that the heart possesses as the work performed and energy expended increases. Therefore, cardiovascular hemodynamics that change during tourniquet application should be managed with individualization. By individualizing the assessment of all components of cardiovascular hemodynamics (cardiac and vascular) during tourniquet application, the CCE, CPO, and Ea parameters provide insights into the heart’s adaptation to changing conditions and impact on energy utilization, contributing to the potential adjustment of treatment [22,32,33,34]. CCE defines hemodynamic performance in relation to energy consumption and should be less than 1, as not all the energy generated during work is fully recovered, and a portion is spent on irregularities [13]. Approaching zero or having a negative value for CCE provides insight into the energy costs of compensatory mechanisms employed by the heart [13].

In our study, the CCE decreased significantly after tourniquet inflation in both groups; however, this change was more pronounced in the GA group. In patients monitored with dP/dt*_max_* to observe the impact of cardiac and vascular changes caused by increased sympathetic tone on LV contractile function, an increase was observed in dP/dt*_max_* in both the groups. The increase in dP/dt*_max_* was parallel to the increased sympathetic tone and consequent increase in Ea. In studies comparing CCE with dP/dt*_max_*, a positive correlation was demonstrated between CCE and dP/dt*_max_* [15,35]. However, in our study, while dP/dt*_max_* increased in both groups, CCE decreased and exhibited inverse changes. For a positive correlation between dP/dt*_max_* and CCE, there must be an increase in the SVI parallel to the increase in dP/dt*_max_*. In our patients, we believe that there was no increase in CCE as there was no increase in SVI during the increase in dP/dt*_max_*. In response to increased left ventricular workload and associated energy consumption (LVESP, contractility), the lack of an increase in SVI may be the reason for the decrease in CCE. Although an increase in venous return is expected after tourniquet inflation, in our patients, this increase was deemed insufficient (due to fasting duration), or the combination of increased venous return and sympathetic tone, along with increased contractility (dP/dt*_max_*), could only compensate for the change in increased afterload, leading to a decrease in CCE. According to our findings, tourniquet inflation decreases cardiac efficiency by increasing contractility and arterial load parameters, regardless of the anesthesia method; however, this negative effect was more pronounced in the GA group.

In the literature, it was reported that in patients under anesthesia, a second pain response emerges approximately 1 h after tourniquet inflation [25,28]. High concentrations of inhalation anesthetics are required to block pain fibers [36]. Despite spinal anesthesia, the mechanism of tourniquet pain formation involves the spread of pain impulses along small myelinated C-fibers [5]. In our study, similar to the literature, a second tourniquet pain response was observed in both patient groups 60–90 min after tourniquet inflation, leading to a remarkable increase in Ea and dP/dt*_max_* values and a decrease in cardiac efficiency. This effect was more evident in the GA group.

Samii et al. reported that after tourniquet release, the redistribution of blood volume to ischemic limbs decreased the preload, leading to the development of hypotension [4]. Townsend et al. associated hypotension following tourniquet deflation with a decrease in preload and peripheral vascular resistance [37]. In our study, an increase was identified in CCE despite a decrease in Ea and dP/dt*_max_* in the GA group after the tourniquet release. The decrease in dP/dt*_max_*, indicating contractility, normally leads to a decrease in SVI, but the decrease in Ea allows the same SVI to be maintained, resulting in an increase in cardiac efficiency.

In the CSEA group, a more stable hemodynamic course resulted in no significant changes in SVI, Ea, dP/dt*_max_*, or CCE values after tourniquet deflation. Despite no significant increase in CCE values after tourniquet deflation in the CSEA group, it remained higher than in the GA group.

This period stands out as a phase in which cardiac efficiency is preserved compared with the tourniquet inflation period, but blood pressure is lower. Therefore, especially in coronary artery disease patients, this period may be crucial, and as indicated in the literature, it could be a time when fatal outcomes may occur [6,7]. Deflation of the tourniquet resulted in fewer hemodynamic fluctuations in the CSEA group than in the GA group, and cardiac function was better preserved.

One of the limitations of our study is the observational nature of the study, which prevented researchers from intervening to correct the changing PRAM parameters (CCE, CPO, dP/dt*_max_*, Ea), and any interventions that could have been made were not evaluated for their effectiveness on PRAM parameters. Another limitation is that clinical follow-up was performed only intraoperatively and there was no postoperative troponin monitoring. More comprehensive prospective randomized controlled trials are needed to generalize these results.

## 5. Conclusions

The use of tourniquets in TKA surgery reduces cardiac efficiency in all patients, regardless of the anesthesia method, and this effect is milder with the CSEA method. While tourniquet inflation reduces cardiac efficiency through changes in aortic impedance and contractility, the improvement in cardiac efficiency upon tourniquet deflation may pose risks due to the potential development of hypotension. The potential development of hypotension may pose a risk for adverse cardiac events ranging from myocardial infarction to death, especially in patients with coronary artery disease.

For these reasons, both preoperative and intraoperative cardiac reserve evaluation should be performed properly in patients undergoing tourniquet-guided TKA. We think that intraoperative cardiac function evaluation of CCE, CPO, dP/dt*_max_*, and Ea parameters obtained by the PRAM method may contribute significantly to the maintenance of hemodynamic stability.

## Figures and Tables

**Table 1:** Patients’ characteristics.

	Total	GA Group	CSEA Group	*p*
Number of Patients, *n* (%)	43 (100.0)	22 (51.2)	21 (48.8)	
Age, years	70 ± 9	67 ± 9	70 ± 9	0.077
Woman, *n* (%)	34 (79.1)	17 (77.3)	17 (81.0)	1.000
BMI, kg m^−2^	30.4 ± 4.8	30.8 ± 5.2	30.0 ± 4.4	0.276
ASA ≥ 2, *n* (%)	38 (88.4)	20 (90.9)	18 (85.7)	0.181
EF (preoperative), %	60 (55–65)	60 (59–65)	60 (55–65)	0.212
Comorbidities, *n* (%)				
HT	31 (72.1)	17 (77.3)	14 (66.7)	0.438
DM	19 (44.2)	9 (40.9)	10 (47.6)	0.658
CAD	13 (30.2)	8 (36.4)	5 (23.8)	0.370
COPD	9 (20.9)	5 (22.7)	4 (19.0)	0.767
CVD	5 (11.6)	4 (18.2)	1 (4.8)	0.345
CRF	2 (4.7)	1 (4.5)	1 (4.8)	1.000
Tourniquet duration, min	96 ± 15	96 ± 17	96 ± 13	0.958

Data are presented as mean ± SD, median [IQR], or number (proportion). Student *t*, Mann–Whitney U and chi-square tests were used for comparisons of groups. GA, general anesthesia; CSEA, combined spinal epidural anesthesia; BMI, body mass index; ASA, American Society of Anesthesiologists; HT; hypertension, DM, diabetes mellitus; EF, ejection fraction; CAD, coronary artery disease, COPD, chronic obstructive pulmonary disease; CVD, cerebrovascular disease; CRF, chronic renal failure.

**Table 2:** Comparisons of PRAM parameters before and after tourniquet.

	Before Tourniquet Inflation (T2)	After Tourniquet Inflation (T3)	*p*	Before Tourniquet Deflation (T8)	After Tourniquet Deflation (T9)	*p*
***In all patients (n = 43)***						
SAP (mmHg)	104 (90 to 121)	135 (117 to 153)	***<0.001***	145 ± 29	113 ± 22	***<0.001***
SVI (mL/m^2^)	42 ± 12	41 ± 12	0.167	42 ± 12	39 ± 12	0.096
Ea (mmHg mL^−1^)	0.91 (0.71 to 1.14)	1.23 (0.96 to 1.60)	***<0.001***	1.18 (1.08 to 1.75)	0.95 (0.81 to 1.37)	***<0.001***
dP/dt*_max_* (mmHg s^−1^)	0.84 ± 0.28	1.08 ± 0.34	***<0.001***	1.22 ± 0.43	0.93 ± 0.40	***<0.001***
CPO (W)	0.68 (0.57 to 0.80)	0.88 (0.74 to 1.11)	***<0.001***	0.95 (0.83 to 1.23)	0.73 (0.57 to 0.84)	***<0.001***
CCE (unit)	0.27 (−0.01 to 0.41)	−0.10 (−0.52 to 0.26)	***<0.001***	−0.12 (−0.43 to 0.17)	0.08 (−0.20 to 0.32)	***0.024***
***In GA group (n = 22)***						
SAP (mmHg)	94 ± 13	136 ± 30	***<0.001***	154 ± 28	106 ± 24	***<0.001***
SVI (mL/m^2^)	40 ± 12	38 ± 11	0.259	38 ± 12	35 ± 11	0.106
Ea (mmHg mL^−1^)	0.80 (0.68 to 1.25)	1.51 (1.15 to 1.67)	***<0.001***	1.58 (1.16 to 2.13)	0.97 (0.81 to 1.61)	***<0.001***
dP/dt*_max_* (mmHg s^−1^)	0.69 ± 0.25	1.06 ± 0.44	***<0.001***	1.28 ± 0.50	0.75 ± 0.28	***<0.001***
CPO (W)	0.60 ± 0.11	0.95 ± 0.27	***<0.001***	1.13 (0.82 to 1.36)	0.65 (0.47 to 0.77)	***<0.001***
CCE (unit)	0.08 ± 0.38	−0.52 ± 0.42	***<0.001***	−0.40 (−0.65 to −0.17)	0.05 (−0.33 to 0.18)	***0.024***
***In CSEA group (n = 21)***						
SAP (mmHg)	121 (107 to 143)	135 (120 to 148)	***<0.001***	132 (118 to 147)	119 (110 to 122)	***<0.001***
SVI (mL/m^2^)	45 ± 11	44 ± 12	0.445	46 ± 11	42 ± 12	0.055
Ea (mmHg mL^−1^)	0.92 (0.82 to 1.05)	1.00 (0.88 to 1.24)	***<0.001***	1.10 (0.91 to 1.22)	0.95 (0.83 to 1.27)	0.118
dP/dt*_max_* (mmHg s^−1^)	1.00 ± 0.22	1.09 ± 0.19	***0.003***	1.16 ± 0.35	1.12 ± 0.42	0.369
CPO (W)	0.85 ± 0.20	0.93 ± 0.27	***0.043***	0.92 (0.81 to 1.01)	0.80 (0.68 to 0.93)	***0.008***
CCE (unit)	0.30 ± 0.18	0.16 ± 0.25	***<0.001***	0.17 (−0.11 to 0.37)	0.28 (0.09 to 0.40)	0.375

Data are presented as mean ± SD, median [IQR], or number (proportion). Paired sample t and Wilcoxon tests were used to compare parameters in T2 and T3 and T8 and T9. GA, general anesthesia; CSEA, combined spinal epidural anesthesia; T2, 10 min after anesthesia induction, which is 1 min before tourniquet inflation; T3, 1 min after tourniquet inflation; T8, 1 min before deflation of the tourniquet; T9, 1 min after deflation of the tourniquet; SAP, systolic arterial pressure; SVI, stroke volume index; Ea, effective arterial elastance (the physiological range at rest is 1.10–1.40 mmHg mL^−1^); CPO, cardiac power output (the physiological range at rest is 0.80–1.20 Watts); CCE, cardiac cycle efficiency (the physiological range is <0 and <1); dP/dt*_max_*, (the physiological range at rest is 0.9–1.3 mmHg s^−1^).

**Table 3:** Comparisons of delta values of PRAM parameters after tourniquet inflation and deflation in GA and CSEA groups.

	After the Tourniquet Inflation (T3 Values Minus T2 Values)			After the Tourniquet Deflation (T9 Values Minus T8 Values)		
	GA (*n* = 22)	CSEA (*n* = 21)	*p*	GA (*n* = 22)	CSEA (*n* = 21)	*p*
Delta-SAP (mmHg)	51 (17 to 59)	10 (5 to 15)	***<0.001***	−48 ± 34	−15 ± 14	***<0.001***
Delta-SVI (mL/m^2^)	−2 ± 9	−1 ± 6	0.626	−3 ± 10	−4 ± 9	0.588
Delta-Ea (mmHg mL^−1^)	0.53 (0.03 to 0.81)	0.16 (0.07 to 0.24)	***0.042***	−0.46 ± 0.43	−0.09 ± 0.29	***0.002***
Delta-dP/dt*_max_* (mmHg s^−1^)	0.40 (0.09 to 0.50)	0.05 (0.01 to 0.21)	***<0.001***	−0.45 (−0.91 to −0.19)	−0.05 (−0.12 to 0.02)	***<0.001***
Delta-CPO (W)	0.35 ± 0.24	0.08 ± 0.17	***<0.001***	−0.41 (−0.65 to −0.25)	−0.09 (−0.27 to −0.01)	***<0.001***
Delta-CCE (unit)	-0.57 ± 0.50	-0.14 ± 0.16	***<0.001***	0.30 (-0.05 to 0.62)	0.03 (-0.09 to 0.32)	0.061

Data are presented as mean ± SD, median [IQR], or number (proportion). Delta changes were calculated with the formula “T3 values—T2 values” and “T9 values—T8 values”. Student *t* and Mann–Whitney U tests were used for comparisons of groups. GA, general anesthesia; CSEA, combined spinal epidural anesthesia; T2, 10 min after anesthesia induction, which is 1 min before tourniquet inflation; T3, 1 min after tourniquet inflation; T8, 1 min before deflation of the tourniquet; T9, 1 min after deflation of the tourniquet; SAP, systolic arterial pressure; SVI, stroke volume index; Ea, effective arterial elastance; CPO, cardiac power output; CCE, cardiac cycle efficiency.

## Data Availability

The datasets for the current study are available from the corresponding author upon reasonable request.
